# First person – Stephanie May

**DOI:** 10.1242/dmm.052455

**Published:** 2025-05-19

## Abstract

First Person is a series of interviews with the first authors of a selection of papers published in Disease Models & Mechanisms, helping researchers promote themselves alongside their papers. Stephanie May is first author on ‘ A precision image-guided model of stereotactic ablative radiotherapy for hepatocellular carcinoma’, published in DMM. She is a Principal Scientific Officer in the lab of Prof. Tom Bird at the Cancer Research UK Scotland Institute, Glasgow, UK, investigating chemo-preventative therapies for hepatocellular carcinoma (HCC) and the prevention of recurrent/metastatic disease following stereotactic ablative radiotherapy (SABR).


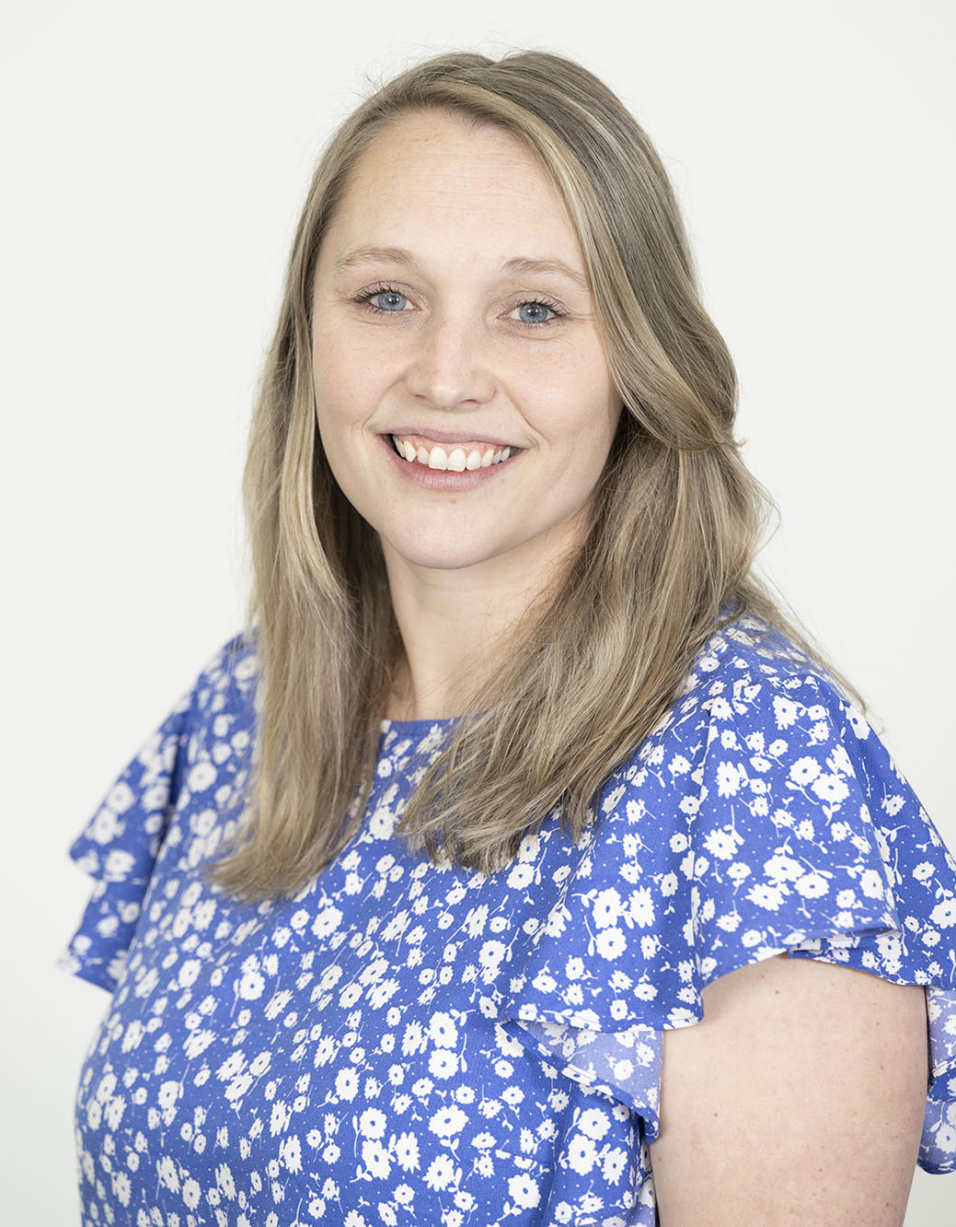


**Stephanie May**

**Who or what inspired you to become a scientist?**

Perhaps spurred on a little too much by watching CSI on TV, I always thought I wanted to become a forensic scientist−there's just something about solving questions/problems/crimes that fascinated me and my favourite subjects in school were always the three sciences, especially Biology. With the intention of becoming a forensic scientist, my A level biology teacher suggested I should consider studying Biochemistry at University as he believed it would open more job opportunities for me. Perhaps unsurprisingly, I absolutely loved all the hands-on lab practicals that were a part of my course. But what really cemented my desire to become a scientist−specifically a cancer researcher−was my final-year dissertation project in Prof. Alan Clarke's group at Cardiff University, UK. I was surrounded by so many amazing scientists, doing really exciting and impactful research and I just knew that this is what I wanted to do and hoped I could also make a difference in the fight against cancer one day.

**What is the main question or challenge in disease biology you are addressing in this paper? How did you go about investigating your question or challenge?**

Liver cancer, specifically hepatocellular carcinoma (HCC), is now one of the biggest contributors to cancer-related deaths worldwide and its prevalence is strongly linked to lifestyle factors. Unfortunately, the majority of patients present with late-stage disease and limited treatment options. Although stereotactic ablative radiotherapy (SABR) has been used in the treatment of other cancer types, it has only recently (in 2020) been approved as a treatment option for a subset of HCC patients, who are not eligible for resection or other local treatments. Some studies report that SABR has good local tumour control, but the majority of patients succumb to recurrent or metastatic disease. To date, there is no suitable preclinical model to study the potential of SABR in HCC treatment, so we aimed to bridge that gap.

As with all preclinical model systems, we wanted to ensure any model we develop reflects the clinical situation as closely as possible. Therefore, we needed to develop a murine model that reflects the subset of patients who would be eligible to receive SABR, implement contrast-enhanced imaging and then − confidently − deliver high-dose radiotherapy specifically to the tumour-limiting radiotherapy exposure to surrounding tissues, as would be done in the clinic.

We have established a syngeneic orthotopic transplant model whereby transplantation of a murine-derived HCC cell line into the murine liver gives rise to an anatomically accurate liver tumour within an immunocompetent setting.

To address these aspects, we have established a syngeneic orthotopic transplant model whereby transplantation of a murine-derived HCC cell line into the murine liver gives rise to an anatomically accurate liver tumour within an immunocompetent setting. In keeping with clinical management and treatment planning, we tested various contrast agent strategies, and have successfully implemented intravenous contrast-enhanced CT imaging in our model to reliably detect and accurately delineate liver tumours. Finally, through immunohistochemistry and longitudinal CT imaging, we were able to show that delivery of 20 Gy SABR induces significant DNA-damage in tumours, reducing the tumour volume, whilst limiting the dose delivered to the surroundings organs and reducing off-target toxicities.

**How would you explain the main findings of your paper to non-scientific family and friends?**

Radiotherapy is a treatment option for many different cancer types but, unfortunately, is associated with a number of nasty side effects. SABR stands for Stereotactic Ablative Body Radiotherapy and is a very precise and powerful form of radiation treatment for cancer. Instead of giving small doses of radiation over many weeks (as in traditional radiotherapy) SABR delivers a few very high doses directly to a small, well-defined tumour. It uses advanced imaging and computer-guided technology to pinpoint the tumour and avoid healthy tissue as much as possible, thereby reducing the nasty side effects. Although SABR has been used for many years in some cancers, it has only recently been approved by the NHS as a treatment option for liver cancer patients. This broadens the treatment options for liver cancer patients but − as it is still only a new therapy for the liver − little is known how best to use this form of treatment, and whether it can be used in combination with other liver cancer therapies. To better understand how liver tumours are likely to respond to SABR and in order to determine which other treatment options can be combined with SABR to improve patient outcomes, we need to do preclinical tests. Animal models are a mainstay of cancer research because they allow scientists to study how cancers develop, grow and respond to treatments in a living system that shares key biological similarities with humans. Therefore, we have developed a mouse model to study the potential of SABR in the treatment of liver cancer. This preclinical model incorporates key elements relevant to the clinical scenario, including computer-guided technology to identify and pinpoint the tumour ready for SABR. Specifically, we were able to show that, by pinpointing the liver tumour through computer-guided imaging, we can specifically deliver high doses of radiotherapy to the tumour, reducing the tumour size post treatment and − whilst minimizing how much radiotherapy is given to the surrounding healthy organs − limiting unwanted side effects. In summary, we have developed a suitable preclinical mouse model that reliably mimics delivery of SABR to liver cancers in human patients. Crucially, this mouse model also allows us to test different therapy combinations, hopefully, improving patient response to SABR in the future.

**What are the potential implications of these results for disease biology and the possible impact on patients?**

This research is the first proof-of-principle preclinical study to investigate the potential of SABR in the treatment of HCC. No preclinical model is perfect; but I believe we have developed a platform that incorporates several aspects of human HCC, including clinical management and treatment planning. Data pertaining to this model have the potential to be integrated with clinical trials of SABR in HCC − to not only demonstrate the human relevance of the model platform but to also explore SABR treatment response biomarkers to improve patient outcomes. I believe that our model will help guide the development of future clinical trials for multimodal HCC therapy and provide a unique opportunity to optimise SABR-inclusive therapy combinations for precision medicine to treat HCC.

**Figure:**
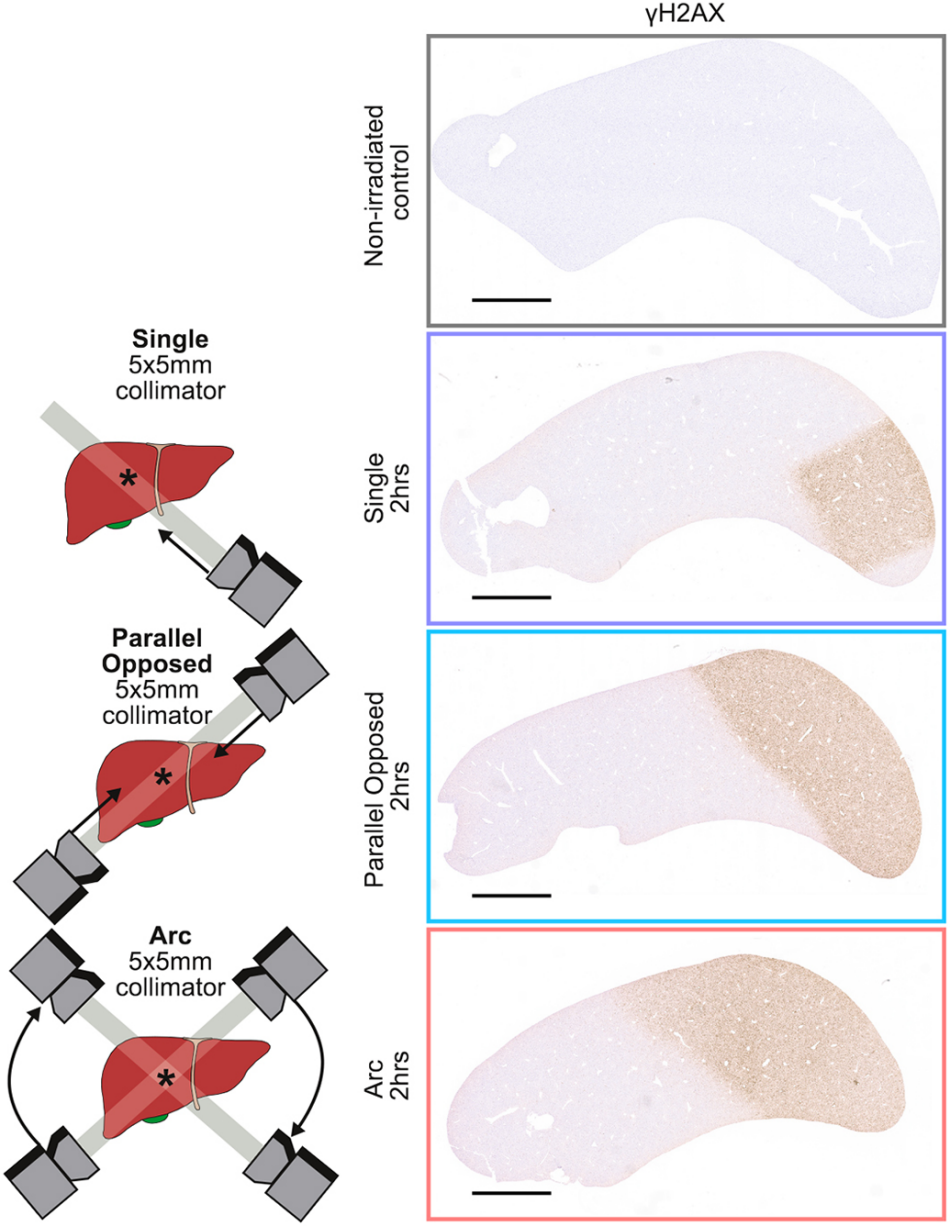
**Stereotactic ablative radiotherapy (SABR) is a highly precise and targeted form of radiotherapy used in the treatment of cancer, including liver cancer.** Delivery of 20 Gee SABR to the liver of wild-type mice by using three different beam configurations − single, parallel opposed and arc − shows the precision of this therapy, indicated by very precise beam-specific immunohistochemistry staining patterns for the DNA-damage marker γH2AX.

**Why did you choose DMM for your paper?**

Our manuscript has been a great example of collaboration between basic scientists, clinicians and radiographers to develop a preclinical platform suitable to investigate the role stereotactic ablative radiotherapy could play in the treatment of HCC in the clinic. Given DMM's emphasis on the development of new disease models, and its translational impact at the interface of basic and clinical science, DMM felt like the perfect fit for our research.

**Given your current role, what challenges do you face and what changes could improve the professional lives of other scientists in this role?**

In my opinion the stages of a research career−from undergraduate and postdoctoral fellowships to the precarious pathway to a permanent position−are inherently unstable. Funding is often short-term and highly competitive; job security is uncertain, and institutional expectations are often opaque, requiring us to spread ourselves too thin to not only strive for good quality, high-impact data − all whilst trying to make our CVs attractive for our future employer. In this environment, I believe mentorship needs to become more than a professional courtesy. Whilst some researchers are lucky to find engaged mentors who will advocate for them, too often, mentorship relies on personal goodwill rather than systemic support. Good mentors need to provide guidance on a broad range of topics including research direction, career strategy, publishing, fostering good working relationships and lab culture. Without that, I believe that early-career researchers are left to reinvent the wheel, often in isolation. I can say that – hand on heart – without the support and mentorship of my PI, I would not be where I am today with my career.

Whilst I think that more mentorship training should be provided for PIs in a career that is highly competitive, we should all strive to contribute to someone else's development and share in their success.

Although PIs often provide strategic guidance and long-term career support, I think it is extremely important to highlight that valuable mentorship can − and should − come from our peers, who offer day-to-day advice, technical expertise and emotional support. Even more-junior colleagues can play a mentoring role by offering fresh perspectives, fostering a culture of mutual learning and encouraging reflection on one's own approaches. This diversity of mentorship sources enriches scientific development and helps build inclusive, resilient research communities where everyone contributes to each other's growth. Therefore, whilst I think that more mentorship training should be provided for PIs in a career that is highly competitive, we should all strive to contribute to someone else's development and share in their success.

**What's next for you?**

Over the past few of years, I have been fortunate enough to secure a couple of CRUK grants to kick start my independent research. I am delighted to say that this publication is my first paper from these independent funds. One of the major roadblocks for early-career researchers has been access to independent funding schemes; however, funders are increasingly supportive of applications from early-career researchers. While I am not technically a group leader, being lead PI on these grants provided me with a unique opportunity to push my independent research whilst also hiring staff and training/mentoring them. So, for me, my focus for the next year or two is to continue training the next generation of scientists and continuing my two research interests−prevention of HCC and multi-modal SABR therapy in HCC−with the view to applying for fellowships. Additionally, I have also just returned to work after a year's maternity leave. So I will be learning to navigate a good work−life balance to continue furthering my longer term research objectives but also enjoying being a mum.

**Tell us something interesting about yourself that wouldn't be on your CV**

Outside of work I love gardening, growing our own vegetables. There is something really satisfying about growing plants from seeds to seeing those vibrant colours on your plate and tasting the fruits of your labour. I just think it is such a rewarding experience that I hope to pass on to our little boy. Living in Scotland, where sunshine is a rarity, gardening isn't always glamourous−but at least we don't have to water the garden too frequently.
